# Evaluation of the Anxiolytic Activity of NR-ANX-C (a Polyherbal Formulation) in Ethanol Withdrawal-Induced Anxiety Behavior in Rats

**DOI:** 10.1155/2011/327160

**Published:** 2010-09-07

**Authors:** L. Mohan, U. S. C. Rao, H. N. Gopalakrishna, V. Nair

**Affiliations:** ^1^Department of Pharmacology, Kasturba Medical College, Mangalore, Karnataka 575001, India; ^2^Department of Pharmacology, All India Institute of Medical Sciences, Ansari Nagar, New Delhi 110029, India

## Abstract

The present study investigates the anxiolytic activity of NR-ANX-C, a standardized polyherbal formulation containing the extracts of *Withania somnifera, Ocimum sanctum, Camellia sinensis*, Triphala, and Shilajit in ethanol withdrawal- (EW-) induced anxiety behavior in rats. Ethanol dependence in rats was produced by substitution of drinking water with 7.5% v/v alcohol for 10 days. Then, ethanol withdrawal was induced by replacing alcohol with drinking water, 12 hours prior to experimentation. After confirming induction of withdrawal symptoms in the alcohol deprived animals, the anxiolytic activity of the test compound in graded doses (10, 20, and 40 mg/kg) was compared to the standard drug alprazolam (0.08 mg/kg) in the elevated plus maze and bright and dark arena paradigms. In our study, single and repeated dose administration of NR-ANX-C reduced EW-induced anxiety in a dose-dependent manner. Even though the anxiolytic activity was not significant at lower doses, NR-ANX-C at the highest dose tested (40 mg/kg) produced significant anxiolytic activity that was comparable to the standard drug alprazolam. Based on our findings we believe that NR-ANX-C has the potential to be used as an alternative to benzodiazepines in the treatment of EW-induced anxiety.

## 1. Introduction

Ethanol has always occupied an important place in the history of Human civilization as a social recreational agent and is the most widely used intoxicating substance in the world [1–3]. When used in low to moderate quantities, it relieves anxiety and fosters a feeling of well being and euphoria. However, excessive alcohol consumption over a prolonged period of time results in the development of alcohol dependence [4]. Subsequently, a decline in the concentration of ethanol in the brain, either due to total cessation of alcohol consumption or a reduction in intake, leads to the emergence of abstinence syndrome [5]. Ethanol withdrawal (EW)/abstinence syndrome is characterized by a constellation of signs and symptoms, with anxiety being a hallmark symptom in humans. Along with this, anxiety is also thought to be the most important negative motivation to revert to alcohol use [6].

Although agents like benzodiazepines are the mainstay for management of anxiety in EW, their use is associated with side effects like development of sedation, drug dependence, and so forth, which limits their usefulness in the clinical setting [7]. Therefore, of late there have been attempts to develop newer agents from Complimentary and Alternative systems of Medicine, as these agents are claimed to be as efficacious as the standard drugs while having minimal side affects [8]. A number of plants like *Ocimum sanctum, Withania somnifera, *and *Camellia sinensis* have been used as components of polyherbal formulations in the Ayurvedic system of medicine for treatment of anxiety disorders. Practitioners of Ayurvedic system of medicine prefer polyherbal formulations over individual agents as the Ayurvedic treaties state that a combination of drugs ensures the potentiation of the therapeutic efficacy of the main drug [9].

The test drug NR-ANX-C is a standardized polyherbal formulation developed by Natural Remedies Pvt. Ltd, Bangalore, India. It is made up of the standardized extracts of *Withania somnifera *(Ashwagandha)*, Ocimum sanctum *(Tulsi)*, Camellia sinensis *(Green Tea), Triphala (made up of fruits of *Emblica officinalis, Terminalia chebula, * and *Terminalia bellericans*), and Shilajit. There are a large number of experimental studies that have evaluated the central activity of these individual plant extracts. *Withania somnifera* [10]*, Ocimum sanctum* [11], and* Camellia sinensis* [12] have been demonstrated to be effective as antistress agents, and *Withania somnifera *[13] and Shilajit have been reported to possess significant anxiolytic activity [14]. Previously, experimental studies carried out in our laboratory have also shown significant anticataleptic [15] and antianxiety [16] activity in NR-ANX-C. Based on this evidence of central activity, we hypothesized that NR-ANX-C could also possess beneficial effects in EW-induced anxiety. In the present study, we have evaluated the anxiolytic effects of NR-ANX-C in EW-induced anxiety. Since anxiety in animals is a subjective state and difficult to quantify [17, 18] we have relied on objective animal models for characterization of this behavioral state.

## 2. Methods

### 2.1. Animals

Male Wistar albino rats weighing 180–200 g from our breeding stock were used in the study. They were housed in groups of three animals per cage and maintained on a 12:12 hour light/dark cycle at an ambient temperature of 25 ± 2°C. The study protocol was approved by Institutional Animal Ethics Committee, Kasturba Medical College, Mangalore, and all experiments were carried out in accordance with “Guidelines for care and use of animals in scientific research (Indian National Science Academy 1998, Revised 2000).”

After acclimatization for seven days, the animals were housed singly in metabolic cages. They were divided into two sets, set one containing six groups and set two containing five groups (*n* = 6). Set one was used for acute study and set two was used for chronic study. One group from set one received water and food *ad libitum* for 10 days and served as the normal control. This group was tested under the same conditions and was used to demonstrate the increase in anxiety behavior after EW in the alcohol-fed animals. The other five groups from set one and all groups from set two received alcohol (7.5% v/v) in drinking water and food *ad libitum *for 10 days [19]. 

### 2.2. Test Drug

The test drug NR-ANX-C is a standardized polyherbal formulation (supplied by Natural Remedies Pvt. Ltd, Bangalore) containing extracts of *Withania somnifera *17 percent (aqueous extract of root: total withanolides 2.1% w/w), *Ocimum sanctum *17 percent (70% alcohol extract of leaves: ursolic acid 2.9% w/w), *Camellia sinensis *33 percent (70% alcohol extract of leaves: total polyphenols 60.1% w/w), triphala 25 percent (aqueous extract of fruits: total tannin 33.5% w/w), and shilajit 8 percent (aqueous extract: fulvic acid 52.6% w/w; humic acid 16.7% w/w).

### 2.3. Experimental Protocol

The standard drug alprazolam and test drug NR-ANX-C were freshly prepared in 1% gum acacia (vehicle) before administration. Five groups of animals were used from each set. Group I received the vehicle and served as the EW control, Group II received alprazolam (0.08 mg/kg body weight), and Groups III, IV, and V received NR-ANX-C in doses of 10, 20, and 40 mg/kg body weight, respectively. For acute study, after alcohol exposure for 10 days, alcohol was withdrawn and substituted with drinking water 12 hours before starting the experiments. Development of EW symptoms in rats was confirmed by using the scoring system defined by Gatch and Selvig [20]. After confirmation of EW symptoms, drugs/vehicle was administered to the respective animals. For chronic study, drugs/vehicle administration was maintained along with alcohol exposure for 10 days. As in acute study, alcohol was withdrawn and substituted with drinking water 12 hours before starting the experiments.

### 2.4. Evaluation of Antianxiety Activity

Sixty minutes after administration of drug/vehicle the animals were sequentially exposed to the following experimental models of anxiety, namely, elevated plus maze and the bright and dark arena. All apparatus were cleaned thoroughly with alcohol after testing each animal in order to mask the odour left by the animal.

### 2.5. Elevated Plus Maze

The elevated plus maze is made of two open arms (50 × 10 cms) and two enclosed arms (50 × 10 × 40 cms) with an open roof, arranged around a central square, such that arms of same type are opposite to each other. The entire maze is raised 50 cm above the ground. In this test, the animal was gently placed in the central square of the maze facing one of the open arms. The number of entries, time spent, and the number of rears in each type of arm (open/closed) was recorded for the duration of 5 min [21].

### 2.6. Bright and Dark Arena

The apparatus consists of an open-top wooden box with two distinct chambers, namely, a dark chamber (20 × 30 × 35 cms) painted black and illuminated with a dim red light, and a bright chamber (30 × 30 × 35 cms) painted white and brightly illuminated with a 100 W white light source placed 17 cms above the box. The two chambers are connected by a small open doorway (7.5 cms) located at floor level in the center of the partition. In this test, the animal was placed in the center of the brightly lit arena. The total number of entries into bright arena, time spent in bright arena, number of rears in bright and dark arena, and the duration of immobility in dark arena were recorded for a period of 5 minutes [22].

### 2.7. Statistical Analysis

Statistical analysis was done by using GraphPad Instat version 3.06 (San Diego, USA). Difference between groups was compared by One-way ANOVA followed by Dunnett's Multiple Comparison test. *P* < .05 was considered significant.

## 3. Results

### 3.1. Acute Study

#### 3.1.1. Elevated Plus Maze

EW produced an increase in anxiety behavior as demonstrated by a decrease in the time spent and number of rears in the open arms and an increase in the time spent and rears in the closed arms by the EW control animals (Table 1). Acute administration of alprazolam reversed this behavior and significantly increased the open arm entries, closed arm entries, total arm entries, percentage of open/total arm entries ratio, time spent in open arms, and rears in open arms as compared to the control (Table 2). Even though NR-ANX-C produced a dose-dependent increase in the anxiolytic behavior, it was significant and comparable to alprazolam only in the highest dose- (40 mg/kg) treated group.

#### 3.1.2. Bright and Dark Arena

EW produced an increase in behavioral anxiety, as seen by a decrease in the time spent in the bright arena and an increase in the number of rears in dark arena and duration of immobility by EW control animals (Table 3). Acute administration of alprazolam decreased the anxiety in the treated animals as is evident by the significant reduction in duration of immobility and increase in time spent in bright arena (Table 4). The test drug NR-ANX-C also produced a dose-dependent reduction in anxiety as demonstrated by increase in entries into the bright arena, time spent in the bright arena, rears, and a decrease in the duration of immobility. However, change in these behavioral parameters was significant only at the highest dose (40 mg/kg) as compared to the control. At the highest dose tested (40 mg/kg), NR-ANX-C was superior to alprazolam in ameliorating EW-induced anxiety behavior in rats.

### 3.2. Chronic Study

#### 3.2.1. Elevated Plus Maze

Chronic administration of alprazolam was effective in reducing EW-induced anxiety behavior in animals as it significantly increased the number of open arm entries, percentage of open arm/total arm entries, time spent in open arms, and number of rears, while reducing the time spent in closed arms, as compared to the control (Table 5). As in acute study, NR-ANX-C produced a dose-dependent anxiolytic effect, which was significant in the higher two doses (20 and 40 mg/kg) as compared to control. At the highest dose tested (40 mg/kg), NR-ANX-C was superior to the standard drug alprazolam in ameliorating EW-induced anxiety behavior in rats.

#### 3.2.2. Bright and Dark Arena

Similar to the results observed in acute study, chronic administration of alprazolam ameliorated EW-induced anxiety behavior in animals as observed by the significant increase in number of entries into the bright arena, time spent in bright arena, rears, and reduction in duration of immobility (Table 6). Chronic administration of NR-ANX-C also produced a dose-dependent inhibition of anxiety behavior. However, as compared to the EW control, the anxiolytic activity was significant only at the higher two doses tested (20 and 40 mg/kg). At the highest dose (40 mg/kg), NR-ANX-C was comparable to the standard drug alprazolam in reversing EW-induced anxiety.

## 4. Discussion

Chronic and excessive ethanol consumption followed by withdrawal results in the development of abstinence syndrome [4, 5]. The most common and prominent feature of alcohol withdrawal is anxiety, which is also considered to be the most important negative motivation to revert to alcohol use [6]. These signs and symptoms of EW have been attributed to the perturbation of central neurotransmitters and ion channel activity. Evidence indicates that during ethanol withdrawal there is an upregulation of excitatory NMDA receptors [23] and a downregulation of inhibitory GABA-A receptors [24]. Therefore, a drug that either facilitates the action of GABA or decreases glutamate activity may be effective in EW-induced anxiety behavior. Since the extent of anxiety is subjective and often difficult to quantify, objective animal models have been used to study the behavioral measures of anxiety during alcohol withdrawal [18]. The elevated plus maze and bright and dark arena are the most commonly employed tests for assessing anxiety like behavior after alcohol withdrawal [25].

In the elevated plus maze, open arms are more fear provoking than the closed arms and the ratio of entries, time spent, and rearing behavior in open arms/closed arms reflects the animal's perception of safety towards closed arms and fearfulness towards open arms [20]. Typical anxiolytic drugs increase the proportion of entries, time spent, rearing in the open arms, and the ratio of open arm to closed arm entries. In the present study, EW control animals showed a reduction in the time spent and rears in the open arms, but increased the time spent in closed arms as compared to normal control (nonalcohol-fed) animals in the elevated plus maze. In the bright and dark arena test, the brightly lit area represents a noxious environmental stressor that inhibits the normal exploratory behavior of rodents and reduction in the number of entries, time spent, and rearing in the bright chamber are considered to be a markers of anxiety [22, 26]. In this model also, the EW control animals demonstrated a marked reduction in the time spent and rearing behavior in the bright chamber as compared to normal control (nonalcohol-fed) animals. Behavioral changes in both these models suggest that EW augmented the behavioral inhibition or anxiety like state in the animals. Both single- and repeated-dose administration of NR-ANX-C produced a dose-dependent anxiolytic effect in these experimental models. At the highest dose tested (40 mg/kg), the anxiolytic activity of NR-ANX-C was comparable to the standard drug alprazolam.

The test drug NR-ANX-C is a standardized polyherbal formulation containing the extracts of *Withania somnifera*,* Ocimum sanctum, Camellia sinensis*, triphala, and shilajit. *Withania somnifera* and its bioactive components have been shown to have GABA mimetic activity [27, 28] and we believe that this property might be primarily contributing towards the anxolytic activity of NR-ANX-C. Additionally, *Withania somnifera* has previously been shown to reduce the levels of tribulin [10], serotonin, and corticotrophin in the brain [29]. Similarly, shilajit has also been shown to reduce the brain levels of serotonin [14]. As an increase in the brain levels of these mediators has been associated with the development of anxiety, a reduction in their levels by these two components could have contributed towards amelioration of the anxiety state. A large number of experimental studies have reported *Ocimum sanctum* to possess anxiolytic activity which has been attributed to it antistressor, cortisol sparing [30, 31], and antioxidant properties [32]. Along with neurotransmitters like GABA and serotonin, adenosine has also been implicated in the development of EW syndrome and adenosine antagonists have been shown to ameliorate this state [33]. *Camellia sinensis* contains methylxanthines which are central nervous system stimulants and adenosine antagonists [34]. We believe that these properties could also have contributed towards the anxiolytic activity of the polyherbal formulation.

**Figure 1:**
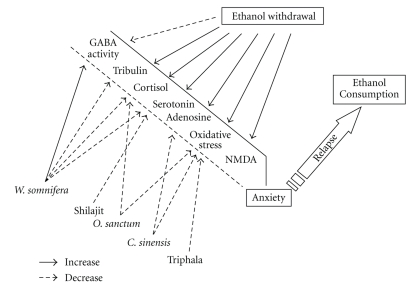
EW decreases GABAergic activity and induces anxiogenic mediators in the brain, thus leading to withdrawal anxiety and a relapse to ethanol consumption. The individual components of NR-ANX-C contribute towards correcting this altered homeostasis, thus alleviating withdrawal anxiety.

As all the individual constituents of NR-ANX-C have been shown to possess antianxiety activity, a question arises as to why a combination has to be used when a higher dose of any single agent can be used to produce a similar effect? The answer to this question lies in Ayurvedic treaties, which state that drugs when used in combination rather than individually ensure potentiation of beneficial effects and amelioration of side effect of the principal drug [9]. This may be true in this case also, as *Withania somnifera, * due to its GABA mimetic activity [27, 28], has sedative properties, and *Camellia sinensis,* due to its methyl xanthine content, has a stimulating effects in brain [35], thus antagonizing the sedative effect. Additionally, *Ocimum sanctum, shilajit, *and* Camellia sinensis* themselves decrease levels of anxiogenic mediators in the brain by different mechanisms, thus potentiating the anxiolytic activity of *Withania somnifera *(Figure 1). In our study, the anxiolytic activity of NR-ANX-C (40 mg/kg) was comparable to that of the standard drug alprazolam in both, elevated plus maze and bright and dark arena paradigms. NR-ANX-C did not produce any significant effect on normal locomotor activity as it did not increase the duration of immobility in the bright and dark arena paradigm. Based on the results of our study, we believe that the polyherbal formulation NR-ANX-C has the potential to be used as an alternative to benzodiazepines in the treatment of ethanol withdrawal.

## Figures and Tables

**Table 1:** EW increases anxiety behavior in elevated plus maze.

Treatment group	No. of entries into		Total arm entries	% of open/total arm entries	Time spent in (s)		No. of rears in	
	Open arms	Closed arms			Open arms	Closed arms	Open arms	Closed arms

Normal control	2.8 ± 0.4	5.3 ± 0.7	8.0 ± 1.0	33.9 ± 1.9	32.6 ± 5.4	207.6 ± 21.8	2.6 ± 0.4	8.1 ± 0.9
EW control	2.3 ± 0.4	5.1 ± 0.9	7.1 ± 1.1	27.9 ± 5.4	18.6 ± 5.0*	270.0 ± 5.5*	1.1 ± 0.5*	10.1 ± 1.4

Values are mean ± SEM; Statistical analysis by One-way ANOVA followed by Dunnett's Multiple Comparison test. *P < .05.

**Table 2:** Acute NR-ANX-C pretreatment reduced EW-induced anxiety behavior in elevated plus maze.

Treatment group	Number of entries into		Total arm entries	% of open/total arm entries	Time spent in (s)		Number of rears in	
	Open arms	Closed arms			Open arms	Closed arms	Open arms	Closed arms

EW control	2.3 ± 0.4	5.1 ± 0.9	7.1 ± 1.1	27.9 ± 5.4	18.6 ± 5.0	270.0 ± 5.5	1.4 ± 0.5	10.1 ± 1.4
Alprazolam (0.08 mg/kg)	6.2 ± 0.6**	7.3 ± 0.2	13.3 ± 0.7**	45.2 ± 2.3*	81.8 ± 12.5**	207.0 ± 18.5*	5.7 ± 0.8**	14.3 ± 1.0
NR-ANX-C (10 mg/kg)	1.8 ± 0.5	4.3 ± 0.9	6.2 ± 1.3	27.2 ± 5.6	29.0 ± 10.7	254.0 ± 12.9	1.7 ± 0.5	7.5 ± 0.7
NR-ANX-C (20 mg/kg)	2.7 ± 0.5	3.7 ± 0.8	6.3 ± 1.3	42.8 ± 4.2	56.0 ± 17.9	235.0 ± 18.5	2.3 ± 0.8	11.5 ± 1.7
NR-ANX-C (40 mg/kg)	7.0 ± 0.6**	8.0 ± 0.6*	15.0 ± 1.1**	48.2 ± 2.3**	73.8 ± 13.2*	215.0 ± 11.1*	8.7 ± 1.2**	13.0 ± 0.8

Values are mean ± SEM; Statistical analysis by One-way ANOVA followed by Dunnett's Multiple Comparison test. *P < .05; **P < .01.

**Table 3:** EW increases anxiety behavior in bright and dark arena.

Treatment group	No. of entries into bright arena	Time spent in bright arena (s)	Rears in bright arena	Rears in dark arena	Duration of immobility (s)
Normal control	2.2 ± 0.6	16.6 ± 1.5	2.5 ± 0.4	9.6 ± 0.7	14.1 ± 1.2
EW control	1.9 ± 0.2	12.4 ± 1.1*	1.0 ± 0.3*	14.5 ± 0.9*	19.8 ± 1.1*

Values are mean ± SEM; Statistical analysis by One-way ANOVA followed by Dunnett's Multiple Comparison test. *P < .05.

**Table 4:** Acute NR-ANX-C pretreatment reduced EW-induced anxiety behavior in bright and dark arena.

Treatment group	Number of entries into bright arena	Time spent in bright arena (s)	Rears in bright arena	Rears in dark arena	Duration of immobility (s)
EW control	1.9 ± 0.2	12.4 ± 1.1	1.0 ± 0.3	14.5 ± 0.9	19.8 ± 1.1
Alprazolam (0.08 mg/kg)	3.3 ± 0.4	28.6 ± 3.1**	2.8 ± 0.5	17.0 ± 2.1	3.5 ± 1.3**
NR-ANX-C (10 mg/kg)	2.3 ± 0.4	17.0 ± 2.8	1.3 ± 0.4	13.3 ± 1.5	3.2 ± 2.1**
NR-ANX-C (20 mg/kg)	2.5 ± 0.3	18.8 ± 4.1	2.7 ± 0.7	17.0 ± 1.9	2.8 ± 1.3**
NR-ANX-C (40 mg/kg)	3.8 ± 0.6**	30.7 ± 3.5**	3.2 ± 0.5*	19.5 ± 2.5	2.7 ± 1.3**

Values are mean ± SEM; Statistical analysis by One-way ANOVA followed by Dunnett's Multiple Comparison test. *P < .05; **P < .01.

**Table 5:** Chronic NR-ANX-C pretreatment reduced EW-induced anxiety behavior in elevated plus maze.

Treatment group	Number of entries into		Total Arm entries	% of open/total arm entries	Time spent in (s)		Number of rears in	
	Open arms	Closed arms			Open arms	Closed arms	Open arms	Closed arms

EW control	1.6 ± 0.4	5.7 ± 0.8	7.4 ± 1.1	19.8 ± 5.1	15.1 ± 5.7	277.0 ± 5.0	1.1 ± 0.4	10.6 ± 1.3
Alprazolam (0.08 mg/kg)	3.8 ± 0.5*	4.8 ± 0.9	8.7 ± 1.1	45.7 ± 4.2*	54.7 ± 15.1*	234.0 ± 15.1*	3.7 ± 0.6*	16.2 ± 1.5*
NR-ANX-C (10 mg/kg)	3.8 ± 0.7*	4.8 ± 0.8	8.7 ± 1.3	44.8 ± 4.2*	32.2 ± 3.4	262.0 ± 3.8	3.0 ± 0.7	10.8 ± 1.7
NR-ANX-C (20 mg/kg)	4.5 ± 0.6*	4.2 ± 0.8	8.7 ± 0.3	53.1 ± 7.9*	45.0 ± 10.0*	245.0 ± 5.7*	3.8 ± 0.8*	11.2 ± 0.9
NR-ANX-C (40 mg/kg)	4.8 ± 0.3*	4.3 ± 0.7	9.1 ± 0.9	52.74 ± 5.6*	49.7 ± 11.1*	242.0 ± 16.2*	4.8 ±1.1*	19.5 ± 0.6*

Values are mean ± SEM; Statistical analysis by One-way ANOVA followed by Dunnett's Multiple Comparison test. *P < .05; **P < .01.

**Table 6:** Chronic NR-ANX-C pretreatment reduced EW-induced anxiety behavior in bright and dark arena.

Treatment group	Number of entries into bright arena	Time spent in bright arena (s)	Rears in bright arena	Rears in dark arena	Duration of immobility (s)
EW control	1.7 ± 0.2	13.9 ± 2.1	1.0 ± 0.3	11.6 ± 1.8	19.6 ± 4.9
Alprazolam (0.08 mg/kg)	3.0 ± 0.2**	25.7 ± 1.5*	3.0 ± 0.2*	20.7 ± 1.6**	0.9 ± 0.4**
NR-ANX-C (10 mg/kg)	2.3 ± 0.2	22.0 ± 3.2	3.0 ± 0.4*	12.8 ± 1.4	4.2 ± 2.9**
NR-ANX-C (20 mg/kg)	2.7 ± 0.3*	27.2 ± 3.3*	3.2 ± 0.3*	13.2 ± 1.3	2.8 ± 0.7**
NR-ANX-C (40 mg/kg)	2.8 ± 0.3*	30.7 ± 3.6**	7.0 ± 0.9**	18.8 ± 2.5*	1.2 ± 4.1**

Values are mean ± SEM; Statistical analysis by One-way ANOVA followed by Dunnett's Multiple Comparison test. *P < .05; **P < .01.
